# Boarding of Mentally Ill Patients in Emergency Departments: American Psychiatric Association Resource Document

**DOI:** 10.5811/westjem.2019.6.42422

**Published:** 2019-07-22

**Authors:** Kimberly Nordstrom, Jon S. Berlin, Sara Siris Nash, Sejal B. Shah, Naomi A. Schmelzer, Linda L.M. Worley

**Affiliations:** * University of Colorado School of Medicine, Department of Psychiatry, Aurora, Colorado; † Medical College of Wisconsin, Department of Psychiatry and Behavioral Medicine, Milwaukee, Wisconsin; ‡ Columbia University Irving Medical Center, Department of Psychiatry, New York, New York; § Harvard Medical School, Department of Psychiatry, Boston, Massachusetts; ¶ Brigham and Women’s Hospital, Department of Psychiatry, Boston, Massachusetts; || University of Arkansas for Medical Sciences-Northwest, Department of Psychiatry, Fayetteville, Arkansas

## Abstract

The treatment of severe mental illness has undergone a paradigm shift over the last 50 years, away from a primary emphasis on hospital-based care and toward community-based care. Some of the forces driving this deinstitutionalization have been scientific and patient-centered, such as better differentiation between acute and subacute risk, innovations in outpatient and crisis care (assertive community treatment programs, dialectical behavioral therapy, treatment-oriented psychiatric emergency services), gradually improving psychopharmacology, and an increased appreciation of the negative effect of coercive hospitalization, except when risk is very high. On the other hand, some of the forces have been less focused on patient needs: budget-driven cuts in public hospital beds divorced from population-based need; managed care’s profit-driven impact on private psychiatric hospitals and outpatient services; and purported patient-centered approaches promoting non-hospital care that may under-recognize that some extremely ill patients need years of painstaking effort to make a community transition.

The result has been a reconfiguration of the country’s mental health system that, at times, leaves large numbers of people without adequate mental health and substance abuse services. Often their only option is to seek care in medical emergency departments (ED) that have not been designed for the needs of mentally ill patients. Increasingly, many of those individuals end up waiting in EDs for appropriate care and disposition for hours or days. This overflow phenomenon has become so prevalent that it has been given a name: “boarding.” This practice is almost certainly detrimental to patients and staff, and it has spawned efforts on multiple fronts to understand and resolve it. When considering solutions, both ED-focused and systemwide considerations must be explored. This resource document provides an overview and recommendations regarding this complex topic.

## INTRODUCTION

### The Scope of the Problem

With “deinstitutionalization” of psychiatric patients in the 1960s, and the advent of managed care starting in the 1980s, the emphasis of caring for persons with mental disorders has shifted away from state-run facilities and toward both in- and out-patient, community-based treatment facilities. This has led to market forces, rather than population indices, driving down the total number of inpatient psychiatric beds. The trend toward fewer beds, which decreases further during periods of economic downturn, has resulted in more psychiatric care taking place in emergency departments (ED) that may be ill-equipped to handle mentally ill patients. When considering solutions, both ED and systemwide considerations must be explored to reduce inappropriate “boarding” of psychiatric patients in the ED and to improve care.

At the core of the problem is the fact that in recent years more and more patients find themselves seeking care for psychiatric illness in EDs. The annual number of all-cause ED visits has continued to increase^1^,^2^ with 6–10% of patients presenting for psychiatric illness and related concerns.^3^,^4^ This is considered a “small but increasing subset” of the ED visit population.^4^ Psychiatric visits weigh heavily on the ED system. They have been found to occupy more time (42% longer than non-psychiatric visits), result in increased inpatient admission (24% vs 12%) and transfer (16% vs 1%), and occupy a higher percentage of self-pay or charity care (22% vs 16%) compared to non-psychiatric visits.^5^ Furthermore, the duration of time spent in the ED is especially long for patients who require transfer to a different facility or who carry a diagnosis of significant mental illness or substance use disorder.^5^

The term “ED boarding” is subject to interpretation, as there is not one agreed-upon definition. Some have described boarding as the situation that occurs when patients remain in the ED for four or more hours after the decision has been made to admit.^6^ Others define it as a stay in the ED exceeding 24 hours.^7^ Nolan and colleagues went further in their definition to an actual description stating: “Boarding describes ED patients whose evaluation is complete and for whom the decision has been made to either admit or transfer, but for whom there is no available bed.”^8^ This is quite similar to the language used by The Joint Commission, which has defined boarding as, “patients being held in the emergency department or another location after the decision to admit or transfer has been made.”^9^ Although the term is used for all patients awaiting hospitalization, the situation is more ominous for patients with psychiatric issues. One survey revealed that 11% of all ED patients were boarded but 21.5% of all psychiatric ED patients were boarded, and odds of boarding for psychiatric patients were 4.78 (2.63–8.66) times higher than non-psychiatric patients.^8^

A 2008 survey of 1400 ED directors by the American College of Emergency Physicians (ACEP) found 79% of the 328 respondents reported having psychiatric patients boarding in their EDs; 55% of ED directors reported boarders on a daily or at least multiple days per week basis; and 62% reported that there are no psychiatric services involved with the patient’s care while they are being boarded prior to their admission or transfer.^6^ Published average boarding times have ranged from 6.8 hours to 34 hours.^10^–^11^ Fundamentally, then, for psychiatric patients “boarding” means spending extensive time in inappropriate locations – whether in the ED on an inpatient medical floor, or in another equally unsuitable place – while awaiting voluntary or involuntary psychiatric hospitalization.^8^

Multiple factors contribute to the ED boarding of psychiatric patients, ranging from large societal challenges and hospital-systems issues to individual patient characteristics. Although the most frequently cited cause of ED boarding is inpatient bed shortages, the problem really starts much farther upstream. Insufficient funding for lower levels of care from basic community clinics to intensive outpatient programs, community crisis stabilization units, and respite services fuels the crisis and leads patients to seek care in emergency settings. Of the respondents to the ACEP survey, 23% replied they have no accessible community psychiatric resources and 59% had no substance abuse or dual-diagnosis patient services available.^6^ Absence of alternative placement options aside from admission is only one of many constraints facing patients.^12^

Other social factors contributing to delays for patients seeking care in the ED may include the lack of insurance or public insurance, hesitation of private hospitals in accepting un/underinsured patients, lack of ambulances willing to provide transport, time spent handling preauthorization from insurance carriers and other managed care hurdles, homelessness, and difficulty in placing patients with severe psychiatric illness burden.^13^–^14^ Added to this public health systems deficit is the inadequate number of state psychiatric inpatient beds due to funding cuts, inpatient unit closures and bed reductions. Delays in discharge for patients already admitted to psychiatric units awaiting limited outpatient services contribute further to the problem.^15^ And, like the larger world of which it is a microcosm, the ED itself often provides a dearth of available mental health resources. There may be no therapeutic milieu, programming, or consistent provider teams such as are available on inpatient psychiatric units.^16^ Indeed, there are often too few or no psychiatric providers at all in emergency settings.^13^ Many times ED personnel are on their own to determine acute treatment plans for significantly ill psychiatric patients. Some emergency providers may harbor concerns about their liability in treating psychiatric patients. 17

This lack of treatment provided to patients psychiatrically boarding is a major concern. As noted previously, 62% of ED medical directors responding to the ACEP survey reported that there are no psychiatric services involved with the patient’s care while he or she is being boarded prior to admission or transfer.^6^ Boarded patients tend to have higher rates of psychotic and personality disorders, and are more likely to require physical restraints/seclusions.^16^ Due to its loud and chaotic nature, the ED environment can exacerbate underlying conditions.^15^ Iatrogenic worsening of symptoms due to suboptimal ED conditions is not uncommon.^15^ Timely, active interventions can reduce patient anxiety, frustration, and agitation, and may even obviate the need for some admissions.^15^

ED boarding carries a high cost burden, affecting the system and patients in a variety of ways. The average monetary cost to an ED to board a psychiatric patient has been estimated at $2,264.^4^ Beyond the direct monetary costs, the system becomes less efficient. In general, ED boarding contributes to reduced ED capacity, decreased availability of emergency staff, longer wait times for all patients in waiting rooms, increased patient frustration, and increased pressure on staff. Psychiatric patients may require increased use of ancillary support (such as security officers or safety attendants), especially if they are agitated and because they have a statistically increased elopement risk.^4^ On the whole for the ED system, boarding results in increased rates of patients who leave without being seen, longer inpatient stays for those admitted, as well as lost hospital revenue and consumption of ED resources.^4^,^8^,^12^,^15^ Providers experience a higher degree of stress related to boarding of patients, resulting in a greater risk of adverse events, and lower levels of reported patient satisfaction.^10^ Emergency physicians and nurses may carry negative attitudes toward psychiatric patients that in turn can affect the treatment they provide and may lead to adverse outcomes.^18^

Many different solutions to the crisis of ED boarding of patients have been proposed. These include increasing resources such as crisis stabilization units, inpatient beds and mental health resources within medical EDs, as well as increasing funding to outpatient mental health services. In addition, expanding the reach of existing psychiatric resources through telepsychiatry and the diversion of patients to regional, specialized psychiatric emergency services that can allow for directed psychiatric care may have great benefit.^19^,^20^ Ultimately, both ED and greater community and systemwide considerations must be explored to reduce ED boarding and improve patient care.

### Potential Solutions: The Role of the Emergency Department

In reality, boarded patients in an ED may not only be awaiting an actualized disposition, but may also be awaiting care. To improve treatment and outcomes of psychiatric patients during the interval before inpatient hospitalization, EDs should consider several unique aspects of this population.

#### Rapid Treatment of Agitation

The etiology of agitation is broad and includes systemic medical, as well as psychiatric, causes. It is unwise to rely on a “single approach” for management. In the six-article *Western Journal of Emergency Medicine* series on Best Practices in the Evaluation and Treatment of Agitation, the American Association for Emergency Psychiatry (AAEP) supported non-coercive de-escalation as the primary intervention, with the goal being to calm, not sedate, the patient.^21^ The calm patient may be better able to participate in care, while the sedated patient may awaken agitated, creating an ongoing cycle. Over-sedation is associated with prolonged ED visits^18^ and potentially compromises care. Verbal de-escalation, as well as targeted medications should be considered in this treatment.^22^,^23^

Some recommend the use of an agitation rating scale as a tool to identify mild agitation and to prompt appropriate treatment.^18^ Several rating scales are available with determination of the right scale for a hospital largely made by ease of use. It is essential to identify underlying medical etiologies precipitating agitation and to treat them appropriately.^24^ Staff including emergency physicians, nurses and hospital security should be provided with regular training on the management of agitation including verbal de-escalation techniques. Similar to “Code Blue” teams, some hospitals have used specially trained teams to aid in de-escalation of highly agitated patients. Even though these teams may exist, training the entire clinical team on proper de-escalation is essential.

#### Minimization of Restraint and Seclusion Use

Physical restraints should be used only as a last resort,^25^ with use limited to the least amount of time necessary. Restraints and seclusion can be quite traumatic for patients, and these interventions raise the risk for medical complications.^26^ They also can negatively affect a patient’s well-being and trust in care.

#### Evaluation of Medical Comorbidities

Rapid identification of medical needs is critical when any patient presents to an ED. For patients with mental illness, this is no exception. Unless there is a long, established history of a psychiatric illness for which the patient presents similarly with each episode, patients with psychiatric symptoms should first be considered to have one or more medical conditions that are contributing to the clinical presentation. Rapid identification is especially important for those patients who present with agitation.^24^ Similarly, because of the importance of not overlooking “medical mimickers” of psychiatric illness, the AAEP’s recently published consensus guidelines urge the psychiatric and ED communities to move away from the generic concept of “medical clearance.” Evaluations specific to the patient’s signs and symptoms should be undertaken, with results clearly communicated between the ED and any receiving facilities.^27^

#### Active Treatment of Psychiatric Illness

For patients who may require a prolonged stay in the ED, active treatment of the underlying illness should be initiated, rather than focusing care solely on mitigating agitation. Treatment can come in multiple forms, such as medication and brief therapies. If the patient is unable to relay information regarding past helpful treatments, obtaining collateral history from family or outside treatment providers can be useful. Short-term therapies may be both efficacious and practical, although they are often overlooked in the busy emergency setting. Even patients who originally present with suicidal ideation may be stabilized by solution-focused, supportive or family therapies, facilitating discharge home or to a lower level of care. EDs may wish to invest in having social workers or other staff receive training in these basic therapies.

#### Implementation of Observation Units

Observation units in the ED, in concert with active treatment, may help patients avoid the need for psychiatric hospitalization. Patients may present as agitated or suicidal if intoxicated or following an extreme psychosocial event such as a break up, the death of a loved one, or the loss of a job. Use of an observation unit, a safe place in which patients can achieve a sober state or work through strong emotions, may also enable discharge to a lower level of care.

#### Active Treatment for Substance Intoxication or Withdrawal

Similar to the need for active treatment of psychosis or suicidality, much can be done to treat substance intoxication or withdrawal in the emergency setting. Intoxicated patients may present as agitated, confused, or out of control. Targeted and timely treatment for agitation and withdrawal is critical, and may be life-saving. Benzodiazepines are the treatment of choice for stimulant intoxication or alcohol withdrawal.^23^

Importantly, the intoxicated patient may also present with suicidality. Some emergency providers may believe that suicidal thoughts occur only in the context of the intoxication. However, patients should be re-evaluated for suicidal ideation once they have cleared from their intoxicated or withdrawal states. Many EDs have protocols for alcohol withdrawal management but less so for other substance withdrawal syndromes. Protocols ensuring proper monitoring and proactive treatment may improve symptoms, decrease total medication requirements, and limit total ED/hospital time. In addition, regardless of a patient’s presentation from substance use, the ED evaluation provides an opportunity to intervene. Motivational interviewing, a well-established effective intervention technique, is simple, takes little time, and may lead to a patient’s interest or willingness for more intensive treatment.

#### Improved Coordination and Communication Around Disposition

As discussed, patients who present in highly agitated or suicidal states may require inpatient psychiatric care; however, there are also times when they may not, if appropriate front-line treatment is provided. When admission to an inpatient facility is required, direct communication between ED and inpatient providers is the optimal way to ensure a successful transfer of care. It is ideal to have a predetermined guide for medical evaluation so that medical stability is achieved prior to transfer. Laboratory testing may be necessary, but should be specifically individualized to the patient and the presentation. Medication may be necessary to allow for calm patient transfer. If it is determined that a patient can safely be discharged to a lower level of care, it is most effective if this is fully arranged in the ED prior to discharge. Optimally, the ED team should provide a thorough hand-off to the outside provider.

#### Other Hospital-Centered Approaches

It is generally agreed that improved access to psychiatric services will result in better patient care and decrease the time to discharge. Unfortunately, six in ten ED directors report that psychiatric services are not available during the boarding period.^6^,^28^ This may be improved by expanding access to psychiatric services through telepsychiatry and integration of care. Telepsychiatry is being more widely used in emergency settings, and many contracts allow for 24-hour availability of psychiatrists as consultants to the ED service. Similarly, healthcare integration is being increasingly introduced into the ED setting. There are several new models that occur locally, allowing for an embedded mental health team including staff psychiatrists to provide consultation either to care teams or directly to patients.

Where possible, improvements in the environment of the emergency setting may have great benefit for patients with psychiatric illness. Boarding in the chaotic, crowded, noisy, and confined spaces of an ED can be anxiety-provoking, distressing, and may potentially exacerbate psychiatric symptoms. The presence of security, continuous observation, and even being forced to wear hospital clothing can lead patients to feel a loss of control that results in an escalation of symptoms.^15^ Mental health emergency room extension areas provide a therapeutic environment more conducive to caring for patients with psychiatric illness. For hospitals with higher volumes, designated psychiatric EDs specialized in emergency psychiatric care may allow for diversion from typical, medical-emergency facilities.^20^

Within hospitals, improvement in the management of patient flow may help to stave off some of the pressures leading to ED boarding. Bed managers or computerized bed management systems may help increase efficiency by managing inpatient capacity. Case managers in the ED can help aid in community disposition. It is incumbent upon hospital leadership to engage in exploring these options to overcome barriers and improve patient care. Finally, data collection and monitoring is essential if progress toward reducing ED boarding and improvement in the provision of care to boarded patients is to be made. This data can be shared with community partners to help determine further strategies for improvement.

### Potential Solutions: Community Efforts

Confronting the ED boarding challenge will require community involvement at the local, state, and ultimately national level.

#### Determine Local Needs

The creation of a taskforce for key stakeholders to convene and coordinate needs for a local area may be an important first step. Stakeholders include dedicated leadership committed to caring for individuals with psychiatric and substance use illnesses. Psychiatric hospitals/units, EDs, crisis centers, mobile crisis services, outpatient mental health clinics, law enforcement, emergency medical services (EMS) groups, group homes, crisis stabilization units, consumer advocates, peer specialists, judges, and local government all constitute stakeholders.

One strategy to determine local needs is to systematically examine each circumstance resulting in ED boarding; this will help to identify precipitants and potential barriers. Causes for ED boarding generally fall into three categories: front-end, ED, and back-end. ED causes have been previously discussed. Front-end causes relate to the spectrum of community-based crisis care. Back-end issues relate more to disposition options and the presence of adequate community resources, including those for severely mentally ill with treatment-resistant or highly complex conditions. Front-end and back-end causes are most closely related to the community. As many patients are un- or underinsured, financial considerations must be clearly understood when dealing with community resources and how funding might be applied. By trending the causes of ED boarding from the front to the back ends, resources can be allocated where they appear most needed, with data collected to evaluate whether the number of boarding patients decreases with such intervention.

#### Focus On Diversion and Coordination

Diversion of patients to preferred resources allows patients to enter the best system for their care. One way of facilitating diversion is by creating an EMS triage system, agreed upon locally, that directs patients to psychiatric hospitals, EDs, and crisis stabilization units, based on criteria. A recent consensus guideline by the AAEP outlines several such protocols.^29^

In addition, providing support to mobile crisis services (ED or psychiatry backup) may help identify resource options for patients in need before they require an ED visit. Checklists can be created such that group homes and nursing facilities can determine whether to engage mobile crisis services or EMS. For diversion to be successful, however, a spectrum of non-emergency levels of care must be created in the community. These may be walk-in centers, respite programs, or crisis stabilization units. In addition, coordination between EDs, mobile crisis, and non-emergency community resources is essential. Clinics, regardless of size, should have true on-call ability to coordinate after-hours care instead of merely recommending that patients with pressing needs on evenings or weekends present to the ED for care. Adequate care coordination resources for patients within the ED are needed to ensure that all patients have viable, timely, follow-up appointments. Rapid access by community mental health providers is essential.

#### State Involvement

State leaders are responsible for allocating Medicaid funds and block grants, and thus are a vital partner in finding workable solutions to the ED boarding dilemma. Efforts need to focus on improved access to care through funding gaps identified in the analysis of boarding cases. This funding should increase the breadth of alternatives to EDs for crisis treatment such as mobile crisis units, crisis stabilization units, 24-hour walk-in clinics, and short-term residential facilities. Within the ED environment, funding can increase accessibility to telepsychiatry. Improved funding should coincide with measurement-based care including metrics and audits to ensure meaningful impact. Improved reimbursement for care with a focus on parity for mental illness, substance use disorders, and intellectual and developmental disorders will be critical.

In addition to providing financial resources, state governments can help to eliminate or safeguard against laws that decrease communication between healthcare providers, especially state laws that are more restrictive than the Health Insurance Portability and Accountability Act (HIPAA) and that effectively block communication between emergency physicians and community mental health centers. Reductions in other undue legal burdens, such as informed consent for emergency telepsychiatry, would also help in increasing access to care within EDs.

#### National Involvement

Government and professional organizations can also play an important role in solving problems related to ED boarding. Efforts should focus on increased access to lower levels of care. Groups should especially focus on developing funding models that support and stimulate growth, and provide sustainability, with particular focus on access to care. Professional psychiatric organizations should engage with emergency medicine professional associations to create joint workgroups to collaboratively address shared concerns regarding care. The newly formed Coalition on Psychiatric Emergencies, in which the American Psychiatric Association and American College of Emergency Physicians are members, is a great start. In addition, national organizations must engage with both government and insurers to solidify parity.

Specific emphasis should be placed on lobbying for fair reimbursement of services, including psychiatric emergency and inpatient services, as care places a financial strain on hospitals, thus providing a disincentive for hospitals to keep units open or add to existing services. Additionally, efforts must be made to reduce the burdensome precertification process, which is unique to psychiatry and adds to delays in admitting or transferring a patient from the ED. Finally, reducing or eliminating out-of-network hospitals for inpatient services will increase available options in some areas. One final consideration is to train more universally on crisis intervention modalities. Training could start as early as medical school, with advanced training in emergency medicine and psychiatry residency programs. This would better ensure that physicians have the appropriate tools to treat the person in a psychiatric crisis.

## CONCLUSION

As ED visits for those with psychiatric illness continue to rise, collective thought and resources must be applied to reduce the boarding of these individuals in EDs. There are several changes that EDs can make to improve the care of patients who arrive at their doors, but ultimately community, state and national efforts will have to be focused on helping to divert patients to lower levels of care and to help ease transition of those in EDs and the inpatient setting back into the community.
